# Errata

**Published:** 1810-05

**Authors:** 


### ERRATA.

In the paging of the first Sheet of this Number, for 273—288, read 353—368.

